# Characterization of Complete Mitochondrial Genome and Phylogeny of Three Echeneidae Species

**DOI:** 10.3390/ani15010081

**Published:** 2025-01-02

**Authors:** Fenglin Wang, Chenghao Jia, Tianxiang Gao, Xingle Guo, Xiumei Zhang

**Affiliations:** 1 Fisheries College, Zhejiang Ocean University, Zhoushan 316022, China; wfl941122@126.com (F.W.); gaotianxiang0611@163.com (T.G.); hermitgxl@163.com (X.G.); 2 School of Marine Sciences, Ningbo University, Ningbo 315211, China; 3 School of Ecology and Environment, Hainan University, Haikou 570228, China; xicheng121@yeah.net

**Keywords:** Echeneidae, mitochondrial genome, high-throughput sequencing, genomic characteristics, phylogenetic analysis

## Abstract

Species of the family Echeneidae are renowned for their capacity to adhere to various hosts using a sucking disc. This study aimed to examine the mitochondrial genome characteristics of three fish species (*Echeneis naucrates*, *Remora albescens*, and *Remora remora*) within the family Echeneidae and determine their phylogenetic relationships. The findings revealed that the mitochondrial genome lengths of the three species were 16,611 bp, 16,648 bp, and 16,623 bp, respectively, containing 13 protein-coding genes (PCGs), 22 transfer RNA genes (tRNAs), two ribosomal RNA genes (rRNAs), and a D-loop region. Most PCGs utilized ATG as the initiation codon, while only *cox I* used the GTG as the initiation codon. Additionally, seven genes employed incomplete termination codons (T and TA). The majority of PCGs in the three species displayed negative AT-skew and GC-skew values, with the GC-skew amplitude being greater than the AT-skew. The Ka/Ks ratios of the 13 PCGs did not exceed 1, demonstrating these species had been subjected to purification selection. Furthermore, only *tRNA-Ser* (GCT) lacked the D arm, while other tRNAs exhibited a typical cloverleaf secondary structure. Bayesian inference (BI) and maximum likelihood (ML) methods were utilized to construct a phylogenetic tree of the three species based on the 13 PCGs. *Remora remora* was identified as a distinct group, while *R. osteochir* and *R. brachyptera* were classified as sister taxa. This study contributes to the mitochondrial genome database of the family Echeneidae and provides a solid foundation for further systematic classification research in this fish group.

## Simple Summary

Species of the family Echeneidae are known for their unique ability to adhere to various hosts using a sucking disc. However, little is known about the mitochondrial genome and evolutionary relationships within this family. This study analyzed the mitochondrial genome characteristics of three species (*Echeneis naucrates*, *Remora albescens*, and *Remora remora*) from the Echeneidae family and their phylogenetic relationships. The results showed that these species share similar mitochondrial features. Additionally, we found that *R. osteochir* and *R. brachyptera* were grouped together as sister taxa. This research provides valuable genetic data that will help improve the classification of the Echeneidae family and offer new insights into their evolutionary history.

## 1. Introduction

The family Echeneidae, belonging to the order Carangiformes, is distributed across various marine habitats, including tropical, subtropical, and temperate waters [1]. The family Echeneidae is comprised of three genera and eight species: *Echeneis* (*E. naucrates* and *E. neucratoides*), *Remora* (*R. remora*, *R. albescens*, *R. australis*, *R. brachyptera*, and *R. osteochir*), and *Phtheirichthys* (*P. lineatus*) [2]. These fish have a unique first dorsal fin that acts as a modified oval sucker-like organ, enabling them to attach to larger marine animals like whales, sharks, dolphins, and turtles [3,4,5]. This hitchhiking behavior enhances access to food resources, reduces transportation costs, and protects against predators [3,6,7]. Echeneidae research has largely focused on several key areas, including bionomics [8], hydrodynamic loading on hosts [6], unique adhesion capabilities [9], genomic studies [4,10,11], and phylogenetic relationships [2,3,12,13,14,15]. Previous studies have classified the family Echeneidae based on morphological and molecular data. However, the phylogenetic relationships among species within the family Echeneidae remain contentious, particularly concerning the affinities among the *R. brachyptera*, *R. osteochir*, and *R. remora*. One hypothesis, based on morphological analysis, proposed that *R. brachyptera* occupies a distinct branch, while *R. osteochir* and *R. albescens* form a cluster [3]. A contrasting view, derived from partial genetic sequencing, suggested that *R. brachyptera* and *R. remora* are more closely related to one another than to *R. osteochir* [12,15]. An alternative hypothesis was that *R. brachyptera* and *R. osteochir* were more closely related than *R. remora* [2,14]. In addition, there is a scarcity of studies focusing on the mitochondrial genome, potentially limiting our understanding of genetic relationships within the Echeneidae at the mitochondrial level.

The mitochondrial genome of fish is a circular, double-stranded DNA molecule [16] characterized by its simple structure, low molecular weight, independent replication, rapid evolution, and maternal inheritance [17,18,19]. Consequently, it is commonly utilized in phylogenetic analysis, species identification, population genetics, and taxonomic research [20,21,22]. Previous investigations on the identification and phylogenetic analysis of fish species have mainly targeted individual mitochondrial genes, such as ribosomal RNA (*12S rRNA* and *16S rRNA*), cytochrome b (*cyt b*), and cytochrome c oxidase I (*cox I*) [23,24,25,26]. However, relying on partial mitochondrial gene sequences has restricted the ability to identify complex genetic evolutionary relationships. In recent years, the development of high-throughput sequencing technology has enabled the acquisition of a wealth of fish mitochondrial genome data, providing new directions and evidence for exploring the genetic characteristics and evolutionary relationships of fish [27,28,29,30].

The present study aimed to characterize the complete mitochondrial genomes of three fish species (*E. naucrates*, *R. albescens*, and *R. remora*) belonging to the family Echeneidae. The mitochondrial genomes were analyzed for their size, nucleotide composition, AT- and GC-skew, codon usage, and selective pressure on 13 PCGs. Furthermore, a phylogenetic tree was constructed using 13 PCGs to determine the phylogenetic position of the family Echeneidae. These findings offer valuable insights into the taxonomic identification and phylogeny of the family Echeneidae and establish a molecular foundation for future genetic studies within this group.

## 2. Materials and Methods

### 2.1. Collection and Identification of Samples

**Figure 1:**
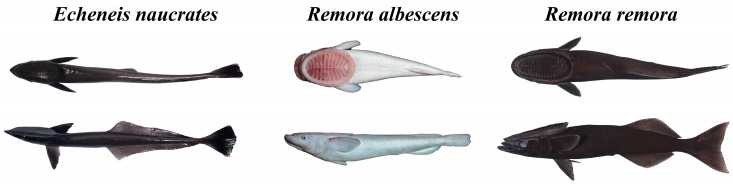
Morphological characteristics of *E. naucrates*, *R. albescens*, and *R. remora*.

The samples of two fish species (*E. naucrates* and *R. albescens*) were collected from the Northern South China Sea in June 2022 (Figure 1), and *R. remora* was obtained in November 2023 from Zhoushan coastal sea, Zhejiang Province, China (Figure 1). Following morphological identification, the muscle sample of one individual of each species was extracted and preserved in 95% ethanol at −80 °C for genomic DNA extraction. All procedures involving handling the samples in this study were performed in strict compliance with the Animal Care Quality Assurance standards of China and Zhejiang Ocean University (Animal Ethics No. 2024150).

### 2.2. DNA Extraction and Whole-Genome Sequencing

Total genomic DNA from muscle tissue was extracted using a DNeasy Blood and Tissue kit (Qiagen, Venlo, the Netherlands) following the manufacturer’s protocol. Subsequently, DNA quality and concentration were evaluated using the NanoDrop 1000 microspectrophotometer and the Qubit fluorometer. DNA samples that passed quality checks were randomly fragmented into approximately 300–350 bp fragments using an ultrasonic crusher. The library preparation process was completed using a NEBNext^®^ Ultra™ II DNA Library Prep Kit for Illumina^®^ (New England BioLabs, Ipswich, MA, USA) in accordance with the manufacturer’s protocol, which involved end-repair, A-tailing, adaptor ligation, purification, and PCR amplification. The constructed library was sequenced using Illumina Nova for PE150 sequencing (OneMore-Tech, Wuhan, China). Finally, sequencing data were processed using fastp v0.23.2 with default parameters to yield high-quality data [31], which included trimming low-quality bases (Q < 20) from read ends, adapter removal, filtering reads with over 40% low-quality bases (Q < 15) or more than 5 ambiguous bases (N), removing reads shorter than 50 bp, and de-duplicating PCR duplicates.

### 2.3. Mitochondrial Genome Assembly, Annotation, and Analysis

NOVOPlasty 2.6.3 software, with its default parameters, facilitated the mitochondrial genome assembly [32]. The *cox I* gene sequences of three fish species were used as seed sequences to assemble the mitochondrial genomes: GU440499.1 (*R. albescens*), GU679459.1 (*R. remora*), and NC_022508.1 (*E. naucrates*). MitoFish was employed to annotate and visualize the complete mitochondrial genome sequence [33]. The base composition, AT- and GC-skew, amino acid usage frequency of protein-coding genes (PCGs), and relative synonymous codon usage (RSCU) were calculated using PhyloSuite v1.2.3 [34]. The tRNAscan-SE v2.0 (http://lowelab.ucsc.edu/tRNAscan-SE/ accessed on 6 June 2024) was employed for tRNA secondary structure prediction. Additionally, DnaSP v6 determined the synonymous (Ks) and non-synonymous (Ka) substitution rates [35], providing further insights into the evolutionary dynamics of the mitochondrial genome.

### 2.4. Phylogenetic Analysis

To analyze the evolutionary relationships of *E. naucrates*, *R. albescens*, and *R. remora*, a phylogenetic tree was reconstructed using 13 PCGs. The mitochondrial genome of *Lates calcarifer* (GenBank ID: NC_007439.1) was utilized as the outgroup, with 18 Carangiformes species (GenBank ID: NC_022707.1, OP057074.2, NC_026718.1, NC_083189.1, NC_082554.1, NC_029421.1, NC_011219.1, NC_028420.1, NC_027183.1, OR582674.1, NC_087992.1, OR668917.1, NC_022508.1, NC_082545.1, NC_022932.1, NC_063497.1, NC_024184.1, and NC_083071.1) selected as the ingroup. PhyloSuite v1.2.3 was employed for the sequence extraction of PCGs from the mitochondrial genomes of all species [34]. An automatic batch alignment of 13 PCG sequences from 22 different species was conducted with MAFFT v 7.471 integrated into PhyloSuite [36]. GBlocks v0.91b was used to trim these sequences using the default settings [37]. Then, all PCGs were concatenated using the connection sequence module in PhyloSuite v 1.2.3. After that, phylogenetic trees were determined by the Bayesian inference (BI) and maximum likelihood (ML) methods. The ML phylogenetic analysis, which utilized the automatic selection option of models, was carried out by IQ-TREE v. 1.6.8 [38] within PhyloSuite v1.2.3 with 1000 bootstrap replicates. For the BI phylogenetic tree, MrBayes v. 3.2.7a integrated into PhyloSuite v1.2.3 was employed [39], utilizing four simultaneous Markov chain Monte Carlo (MCMC) algorithms. Two parallel runs of 2,000,000 generations were executed, with sampling occurring every 1000 generations. The initial 25% of data were discarded as burn-in. Subsequently, the phylogenetic trees were displayed using the online tool iTOL v 6.9 (https://itol.embl.de/) (accessed on 13 June 2024).

## 3. Results

### 3.1. The Base Composition and Structure of the Mitochondrial Genome

**Figure 2:**
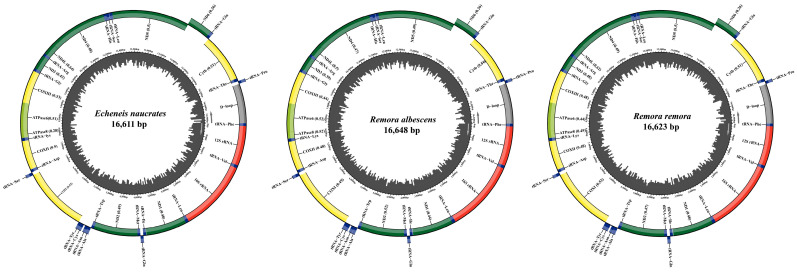
Circular map of the mitochondrial genome of *E. naucrates*, *R. albescens*, and *R. remora*.

The mitochondrial genomes of *E. naucrates*, *R. albescens*, and *R. remora* were 16,611 bp, 16,648 bp, and 16,623 bp in length, respectively (Figure 2). These three fish species possessed circular, closed, double-stranded mitochondrial genomes containing a total of 37 genes. These genes consisted of 13 PCGs (*atpase 6*, *atpase 8*, *cyt b*, *cox I-III*, *nd 1–6*, and *nd4L*), 22 tRNA genes, and two rRNA genes (*12S rRNA* and *16S rRNA*) in addition to a D-loop region (Tables S1–S3). Notably, one PCG (*nd6*) and eight tRNA genes were situated on the light strand (L-strand), while the remaining 28 genes were located on the heavy strand (H-strand).

The mitochondrial genomes of *E. naucrates*, *R. albescens*, and *R. remora* were analyzed, revealing five overlapping regions and 10 intergenic spacer regions in *E. naucrates* and five overlapping regions and 11 intergenic spacer regions in *R. albescens* and *R. remora* (Table S4). The total lengths of overlapping regions were 24 bp in three fishes, while the total lengths of intergenic spacer regions were 65 bp in *E. naucrates*, 68 bp in *R. albescens*, and 67 bp in *R. remora*. The longest overlapping region, spanning 10 bp, was identified between *atpase* 8 and *atpase* 6 in all three fishes. Additionally, the largest intergenic spacer regions, 37 bp in *E. naucrates* and 36 bp in *R. albescens* and *R. remora*, were found between tRNA-Asn and tRNA-Cys.

The nucleotide compositions of the mitochondrial genomes of the three fish species ranged from 28.1% to 29.3% for T, 28.3% to 30.3% for A, 25.4% to 27.6% for C, and 15.0% to 16.2% for G (Tables S5–S7). The A + T content in the mitochondrial genomes of these fish species was higher, ranging from 56.7% to 59.6%, compared to the G + C content, which ranges from 40.4% to 43.4%. This indicated a clear preference for AT base pairs. Furthermore, the AT-skew values in the mitochondrial genomes of *E. naucrates* and *R. albescens* were positive (0.016 and 0.012), while the GC-skew values were negative (−0.257 and −0.276). This indicated that A is more abundant than T, and C is more common than G in these species. On the other hand, the AT-skew value (−0.002) and GC-skew value (−0.253) of *R. remora* were both negative, indicating a greater abundance of T and C in comparison with A and G.

### 3.2. Protein-Coding Genes and Codon Usage

**Figure 3:**
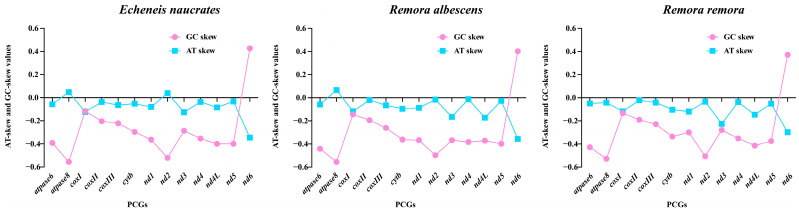
AT-skew and GC-skew values in the protein-coding genes (PCGs) of the mitochondrial genome of *E. naucrates*, *R. albescens*, and *R. remora*.

The complete sequence length of 13 PCGs in the mitochondrial genomes of *E. naucrates*, *R. albescens*, and *R. remora* was 11,418 bp, constituting 68.7%, 68.6%, and 68.7% of their mitochondrial genomes, respectively. Among three fish species, 12 PCGs were situated on the light strand (L-strand), with exclusively *nd6* found on the heavy strand (H-strand). Most PCGs exhibited negative AT-skew and GC-skew values, indicating an elevated content of T and C (Figure 3). Notably, the GC-skew was consistently greater than the AT-skew, with the most significant differences observed in the *nd6* gene across all three species. Twelve PCGs used ATG as the start codon, and only *cox I* started with GTG. Termination codons were identified in the PCGs of three fish species, with *cyt b*, *cox II*, *nd3*, and *nd4* ending with a single T, while *atpase 6*, *nd2*, and *cox III* utilized TA as a termination codon. *Echeneis naucrates* and *R. albescens* exhibited the TAA termination codon in six PCGs (*atpase 8*, *cox I*, *nd1*, *nd4L*, *nd5*, and *nd6*), whereas *R. remora* only used TAA termination codon in five PCGs, with *nd6* employing the TAG termination codon.

**Figure 4:**
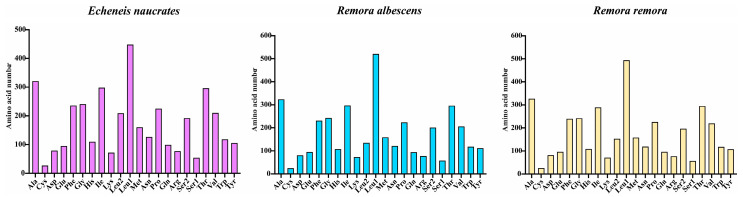
The number of amino acids in the mitochondrial genome of the *E. naucrates*, *R. albescens*, and *R. remora*.

**Figure 5:**
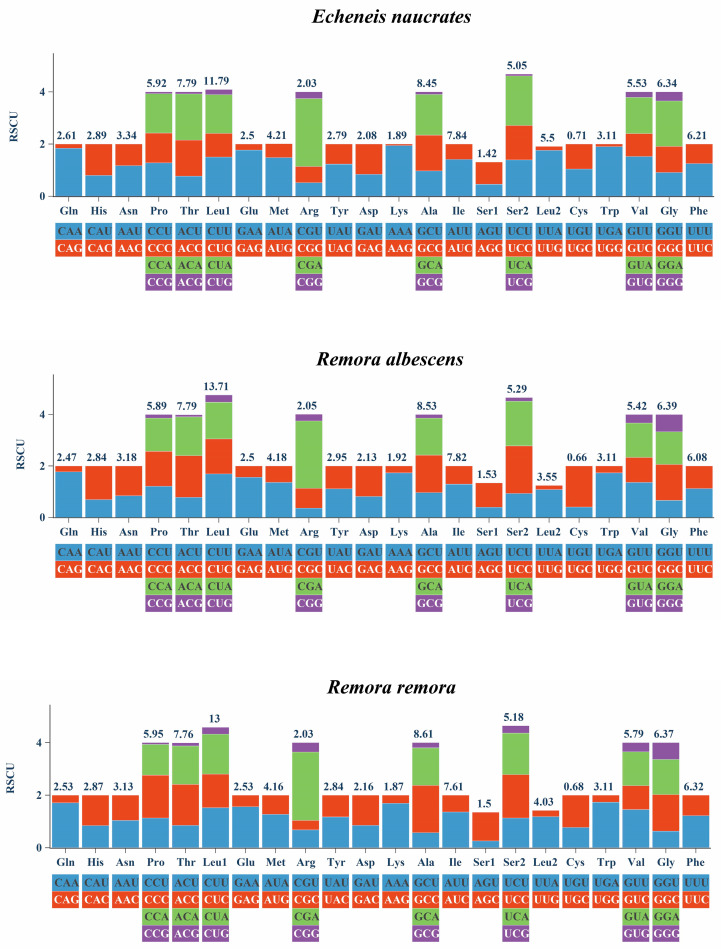
Relative synonymous codon usage (RSCU) of the mitochondrial genome of *E. naucrates*, *R. albescens*, and *R. remora*.

Figure 4 and Figure 5 present the amino acid usage and RSCU values of PCGs in the mitochondrial genomes of three fish species. All three fish species encoded 3800 amino acids within mitochondrial genomes. Among these, Leu was the most frequently utilized amino acid (11.79–13.71%), while Cys was the least utilized (0.66–0.71%). The most commonly used codons include AUU (Ile), UUA (Leu2), CUU (Leu1), and CUA (Leu1).

### 3.3. Selective Pressure Analysis

**Figure 6:**
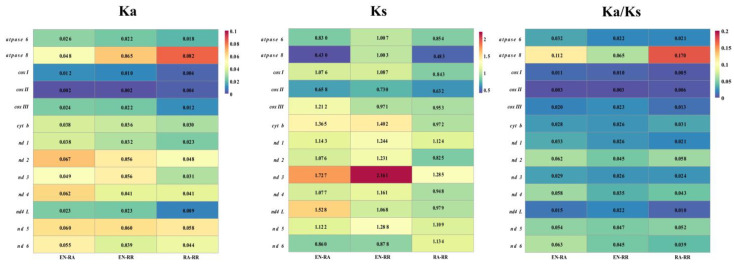
The Ka, Ks, and Ka/Ks values for each protein-coding gene (PCG) in pairs of mitochondrial genomes of *E. naucrates* (EN), *R. albescens* (RA), and *R. remora* (RR).

The ratio of Ka to Ks (Ka/Ks) was utilized to assess the selective pressure on 13 PGCs across three fish species. The Ka/Ks ratios for all PGCs were consistently below 1 (Figure 6), suggesting that strong purifying selection was operating on these genes in the three fish species. Notably, *atpase 8* exhibited the highest Ka/Ks value in *R. albescens* and *R. remora*, indicating a potentially faster rate of evolution compared to other mitochondrial PCGs. Conversely, *cox I* and *cox II* displayed the lowest Ka/Ks values among the three fish species, implying significant selection pressure and slower evolutionary rates for these genes.

### 3.4. Transfer RNAs, Ribosomal RNAs, and D-Loop Region

The sizes of the 22 tRNA genes in the mitochondrial genomes of *E. naucrates*, *R. albescens*, and *R. remora* ranged from 65 to 76 bp, 65 to 75 bp, and 65 to 75 bp, with total lengths of 1549 bp, 1553 bp, and 1552 bp, respectively (Tables S1–S3). All three fish species had three tRNAs on the light strand (L-strand) and fourteen tRNAs encoded on the heavy strand (H-strand). The A + T content (54.5–56.7%) of the tRNAs was greater than the C + G content (43.4–45.5%), indicating a preference for A + T. Furthermore, the tRNAs exhibited positive values for AT-skew (0.019–0.036) and GC-skew (0.032–0.051), suggesting a bias towards A and G. Additionally, the secondary structures of all tRNAs in the three fish species displayed a typical cloverleaf structure, with only *tRNA-Ser* (GCT) lacking the DHU arm (Figure S1).

The two rRNA genes of *E. naucrates*, *R. albescens*, and *R. remora* were all encoded in the heavy strand (H-strand). The lengths of the *12S rRNA* were 948, 947, and 950 bp, respectively, while the lengths of the *16S rRNA* were 1705, 1712, and 1707 bp, respectively (Tables S1–S3). The *12S rRNA* gene was found situated between *tRNA-Phe* and *tRNA-Val*, whereas the *16S rRNA* gene was positioned between *tRNA-Val* and *tRNA-Leu*. The overall A + T content (55.4–57.5%) of the two rRNAs was higher than the C + G content (42.5–44.6%) in the three fish species. Furthermore, both rRNAs exhibited positive AT-skew values (0.182–0.206) and negative GC-skew values (−0.082–−0.065), indicating a bias towards A and C in the tRNAs.

The D-loop regions of *E. naucrates*, *R. albescens*, and *R. remora* were situated between tRNA-Pro and tRNA-Phe, with lengths measuring 940, 964, and 943 bp, respectively (Tables S1–S3). Across all three species, the D-loop region exhibited a noticeable A + T pattern, with an A + T content ranging from 67.1% to 67.9%. Additionally, the AT-skew values were positive (0.001–0.019), while the GC-skew values were negative (−2.15–−2.04).

### 3.5. Phylogenetic Relationships

**Figure 7:**
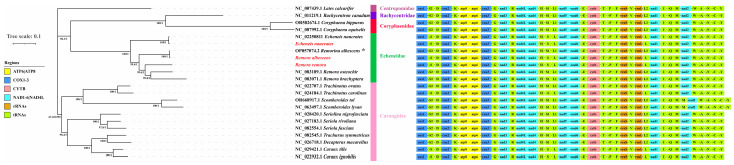
The phylogenetic tree reconstructed from 13 PCGs of *E. naucrates*, *R. albescens*, and *R. remora* using Bayesian inference (BI) and maximum likelihood (ML) methods. The numbers at the nodes are the bootstrap support values (left) and Bayesian posterior probabilities (right). * The genus *Remorina* was attributed to the genus *Remora*.

Phylogenetic trees of *E. naucrates*, *R. albescens*, and *R. remora* were built using ML and BI methods based on the 13 concatenated PCGs. The results demonstrated that the phylogenetic trees constructed through the ML and BI methods exhibited a consistent topological structure (Figure 7). The phylogenetic trees were divided into two main branches. The families Echeneidae, Rachycentridae, and Coryphaenidae are grouped together, while the family Carangidae is a separate entity. The genera *Echeneis* and *Remora*, both belonging to the family Echeneidae, were observed to constitute two discrete clades. In the genus *Remora*, *R. remora* was identified as a distinct branch, while *R. osteochir* and *R. brachyptera* were classified as sister taxa.

## 4. Discussion

In this study, we obtained the complete mitochondrial genomes of *E. naucrates*, *R. albescens*, and *R. remora*, with lengths of 16,611 bp, 16,648 bp, and 16,623 bp, respectively. These lengths were consistent with those reported for other fish species [10,29,30,40]. Notably, this study marked the first report of the mitochondrial genome of *R. remora*. The mitochondrial genomes of all three species exhibited a clear preference for AT bases and an anti-G bias, which was consistent with the base composition of most bony fishes [29,41,42,43]. Various factors can affect the base preferences of the mitochondrial genome, including genetic mutations resulting from selective pressures during species evolution, stochastic genetic drift, and horizontal gene transfer [44,45]. Mitochondrial genes located on the heavy strand (H-strand) were more susceptible to hydrolysis and oxidation, as previously observed [46]. Interestingly, only the *nd6* gene was identified on the light strand (L strand) in our study, suggesting its relative stability and specificity. This phenomenon had been previously documented in other fish species [30,47]. In this study, we examined the mitochondrial genomes of three fish species exhibiting both overlapping and intergenic regions. The longest overlapping region, spanning 10 bp, was located between the *atpase* 8 and *atpase* 6 genes. The overlap between *atpase* 8 and *atpase* 6 is a common feature in fish mitochondrial genomes, observed across a wide range of species [48,49,50]. Such gene overlaps contribute to the compactness of the mitochondrial genome and enhance transcriptional efficiency [43,51], thereby improving environmental adaptability. The total lengths of the intergenic spacer regions were 65 bp in *E. naucrates*, 68 bp in *R. albescens*, and 67 bp in *R. remora*. Variations in the lengths of intergenic spacers directly impact the overall size of the mitochondrial genome [43]. Specifically, *R. albescens* possessed the longest intergenic spacer regions, resulting in a relatively larger mitochondrial genome. Among the three species, the largest intergenic spacer region was found between tRNA-Asn and tRNA-Cys, measuring 36 to 37 bp. This region serves as the origin of replication for the L-strand (OL), a critical element for the replication of mitochondrial DNA [52].

The analysis of the start and stop codons’ usage in the mitochondrial genomes of three fish species yielded significant insights into their translational regulation and evolutionary adaptation. In this study, it was observed that 12 PCGs utilized ATG as the start codon, while the *cox I* gene uniquely employed GTG. This finding was consistent with previous studies on other bony fishes [30,40,48,49]. The prevalence of ATG as a start codon suggested its superior efficiency and preference during transcription and translation initiation [53]. The common usage of GTG as a start codon in the *cox I* gene may indicate specific selective pressures or functional requirements unique to these species. The differing utilization of stop codons between species served to illustrate the intricacy of mitochondrial genome evolution. Incomplete codons T and TA were identified in all three species, which was consistent with known characteristics of fish mitochondrial genomes [49,54]. These incomplete codons can be transformed into complete stop codons through post-transcriptional polyadenylation modifications [55]. In addition, the *nd6* gene used TAA as a stop codon in *E. naucrates* and *R. albescens*, while *R. remora* employed TAA as a termination codon. This suggested that the intact termination codons, TAA and TAG, exhibited species-specific patterns. The differences in stop codon utilization among these closely related species emphasize the dynamic evolution of mitochondrial genomes and their potential to reflect ecological and physiological adaptations [16].

The three fish species included in this study all exhibited negative AT-skew and GC-skew values in PCGs, indicating a higher prevalence of T and C nucleotides. Interestingly, GC-skew consistently surpassed AT-skew, with the *nd6* gene exhibiting the greatest fluctuation in AT/GC-skew values in all three species. The *nd6* subunit, encoded by mitochondrial DNA, plays a crucial role in mitochondrial complex I by contributing to electron transfer from NADH to ubiquinone and aiding in proton translocation across the inner mitochondrial membrane [56]. This function is essential for establishing the proton gradient necessary for ATP synthesis. It was suggested that the selection and mutation pressure associated with the respiratory metabolism of this gene may differ significantly from other genes.

Amino acid usage and RSCU were analyzed in the mitochondrial genomes of three fish species. The results revealed that Leu was the most frequently utilized amino acid, while Cys was the least common. This distribution highlighted a preference for leucine, a branched-chain amino acid (BCAA) known for its impact on mitochondrial function through the mTOR and Opa-1 signaling pathways [57]. Additionally, dietary incorporation of leucine was found to enhance fish humoral immunity and antioxidant capacity [58]. The predominant codons, such as AUU (Ile), UUA (Leu2), CUU (Leu1), and CUA (Leu1), further emphasize the ubiquity of the leucine codon. The utilization patterns of these codons, which may be related to the natural selection of species [59], could provide insights into the origin and evolution of species within the family Echeneidae.

The Ka/Ks ratio is commonly used to assess the selection pressure and evolutionary relationship between homogeneous or heterogeneous species at the molecular level [60]. When Ka/Ks < 1, it suggests negative or purifying selection; when Ka/Ks = 1, it signifies neutral mutations; and when Ka/Ks > 1, it denotes positive or diversifying selection [30]. In mitochondrial genomes, Ka/Ks ratios are generally found to be less than 1, reflecting the strong purifying selection typically acting on these genes due to their essential and conserved functions [61,62]. In this study, all Ka/Ks ratios of 13 PCGs in three fish species were below 1, indicating that these genes were subjected to significant purifying selection to maintain their functional integrity. Among the 13 PCGs, *atpase 8* exhibited the highest Ka/Ks value, with comparable results observed in other fish species [49,54]. This suggested a relatively fast evolutionary rate and potential adaptive changes. Conversely, *cox I* and *cox II* had the lowest Ka/Ks values, indicating strong purifying selection and slower evolutionary rates. These results emphasized the varying selective pressures acting on different mitochondrial genes, reflecting their functional significance and evolutionary dynamics.

A comparative examination of the secondary structure of tRNAs in the mitochondrial genomes of three fish species revealed that the tRNAs retained the typical clover-like structure, except *tRNA-Ser* (GCT) lacking the DHU arm. This phenomenon was prevalent among teleost fish and represents a common feature of fish mitochondrial genomes [29,30,47,49]. Nevertheless, the absence of this structure can be compensated for by subsequent adjustments to the ribosome’s structural form and function, ensuring its continued ability to carry and translocate amino acids [63]. In addition, the *12S rRNA* and *16S rRNA* genes in the three fish species were encoded on the heavy strand, separated by *tRNA-Val*, and positioned between *tRNA-Leu* and *tRNA-Phe* genes. The D-loop region in the mitochondrial genomes of these species exhibited the highest A and T content (67.1–67.9%), indicating the presence of AT-rich regions that were crucial for mitochondrial genome replication and transcription initiation.

In this study, we conducted a phylogenetic analysis of three species in the family Echeneidae using 13 PCGs through ML and BI methods. The results indicated that the genera *Echeneis* and *Remora* within the family Echeneidae form two distinct lineages. This finding was consistent with previous studies [14,15]. O’Toole has previously attributed genus *Remorina* to the genus *Remora* [3]. In the present study, we found that *R. albescens* and *Remorina albescens* clustered together as a single branch, thereby confirming this view. Therefore, a taxonomic revision of the species within the genus *Remora* is essential for laying a solid foundation for in-depth systematic classification studies of genus *Remora* and, more broadly, the family Echeneidae. Our results identified *R. remora* as a distinct branch, with *R. osteochir* and *R. brachyptera* classified as sister taxa, corroborating the findings of Kenaley et al. (2019) and Santini et al. (2015) [2,14]. Our research provides the first comprehensive analysis of the mitochondrial genome and phylogenetics of the family Echeneidae. However, the positions of *P. lineatus*, *R. australis*, and *E. neucratoides* remain uncertain due to limited mitochondrial genome data. To refine the phylogeny of the major species of the family Echeneidae, more sampling of these species is needed. Therefore, future studies should aim to resolve the intricate relationships within the family Echeneidae by integrating comprehensive evidence from molecular and morphological characteristics.

## 5. Conclusions

This study analyzed the mitochondrial genomes and phylogenetic relationships of three Echeneidae species (*E. naucrates*, *R. albescens*, and *R. remora*). Among them, the mitochondrial genome of *R. remora* is the first reported. The mitochondrial genomes of all three species were found to consist of 37 genes, including two rRNA genes, 22 tRNA genes, 13 PCGs, and a D-loop region. The size of the mitochondrial genomes, nucleotide composition, and codon usage are consistent with those reported for other teleost fish. Selective pressure analysis demonstrated that all mitochondrial PCGs of the three fish species were subjected to purifying selection, with the *atpase 8* gene exhibiting the highest evolutionary rate and the lowest observed for the *cox I*/*cox II* genes. Furthermore, the phylogenetic analyses based on 13 PCGs using ML and BI methods revealed *R. remora* as a distinct branch, while *R. osteochir* and *R. brachyptera* were classified as sister taxa. These findings provide fundamental molecular data and reference for the classification and phylogenetic relationships within the family Echeneidae.

## Data Availability

The complete mitogenomes generated during this study were uploaded to GenBank with accession numbers PP994680, PP994681, and PP994682, respectively.

## Supplementary Materials

The following supporting information can be downloaded at https://www.mdpi.com/article/10.3390/ani15010081/s1. Figure S1: Predicted secondary structures of *tRNA-Ser* (GCT) gene in the mitochondrial genome of *E. naucrates*, *R. albescens*, and *R. remora*.; Table S1: Mitochondrial genome composition and characteristics of *E. naucrates*; Table S2: Mitochondrial genome composition and characteristics of *R. albescens*; Table S3: Mitochondrial genome composition and characteristics of *R. remora*; Table S4: Sequence of the mitochondrial genome intergenic region in three species; Table S5: The base composition of *E. naucrates* mitochondrial genome; Table S6: The base composition of *R. albescens* mitochondrial genome; Table S7: The base composition of *R. remora* mitochondrial genome.

## References

[1] Smith J.L.B., Smith M.M., Heemstra P.C. (1986). Smiths’ Sea Fishes.

[2] Kenaley C.P., Stote A., Ludt W.B., Chakrabarty P. (2019). Comparative Functional and Phylogenomic Analyses of Host Association in the Remoras (Echeneidae), a Family of Hitchhiking Fishes. Integr. Org. Biol..

[3] O’Toole B. (2002). Phylogeny of the species of the superfamily Echeneoidea (Perciformes: Carangoidei: Echeneidae, Rachycentridae, and Coryphaenidae), with an interpretation of echeneid hitchhiking behaviour. Can. J. Zool..

[4] Gao T., Liu K., Liu Q., Wang D. (2024). An improved chromosome-level genome assembly and annotation of *Echeneis naucrates*. Sci. Data.

[5] Cressey R.F., Lachner E.A. (1970). The parasitic copepod diet and life history of diskfishes (Echeneidae). Copeia.

[6] Beckert M., Flammang B.E., Anderson E.J., Nadler J.H. (2016). Theoretical and computational fluid dynamics of an attached remora (*Echeneis naucrates*). Zoology.

[7] Muir B., Buckley R. (1967). Gill ventilation in Remora remora. Copeia.

[8] Wang Y., Yang X., Chen Y., Wainwright D.K., Kenaley C.P., Gong Z., Liu Z., Liu H., Guan J., Wang T. (2017). A biorobotic adhesive disc for underwater hitchhiking inspired by the remora suckerfish. Sci. Robot..

[9] Flammang B.E., Kenaley C.P. (2017). Remora cranial vein morphology and its functional implications for attachment. Sci. Rep..

[10] Musika J., Phinchongsakuldit J. (2015). Live sharksucker *Echeneis naucrates* (Linnaeus 1758) mitochondrial genome: The first report of Echeneidae complete mitochondrial genome. Mitochondrial DNA.

[11] Zhou C., Liu Q., Qu Y., Qiao Y., Gao T., Wang D. (2024). The first chromosomal-level genome assembly and annotation of white suckerfish *Remora albescens*. Sci. Data.

[12] Gray K.N., McDowell J.R., Collette B.B., Graves J.E. (2009). A Molecular Phylogeny of the Remoras and their Relatives. Bull. Mar. Sci..

[13] Friedman M., Johanson Z., Harrington R.C., Near T.J., Graham M.R. (2013). An early fossil remora (Echeneoidea) reveals the evolutionary assembly of the adhesion disc. Proc. R. Soc. B..

[14] Santini F., Carnevale G. (2015). First multilocus and densely sampled timetree of trevallies, pompanos and allies (Carangoidei, Percomorpha) suggests a Cretaceous origin and Eocene radiation of a major clade of piscivores. Mol. Phylogenet. Evol..

[15] Glass J.R., Harrington R.C., Cowman P.F., Faircloth B.C., Near T.J. (2023). Widespread sympatry in a species-rich clade of marine fishes (Carangoidei). Proc. R. Soc. B..

[16] Boore J.L. (1999). Animal mitochondrial genomes. Nucleic Acids Res..

[17] Yang Z., Nielsen R., Hasegawa M. (1998). Models of amino acid substitution and applications to mitochondrial protein evolution. Mol. Biol. Evol..

[18] Zardoya R., Meyer A. (1998). Cloning and characterization of a microsatellite in the mitochondrial control region of the African side-necked turtle, *Pelomedusa subrufa*. Gene.

[19] Caccone A., Gentile G., Burns C.E., Sezzi E., Bergman W., Ruelle M., Saltonstall K., Powell J.R. (2004). Extreme difference in rate of mitochondrial and nuclear DNA evolution in a large ectotherm, Galápagos tortoises. Mol. Phylogenet. Evol..

[20] Shao R., Barker S.C. (2007). Mitochondrial genomes of parasitic arthropods: Implications for studies of population genetics and evolution. Parasitology.

[21] Keith P., Lord C., Lorion J., Watanabe S., Tsukamoto K., Couloux A., Dettai A. (2011). Phylogeny and biogeography of Sicydiinae (Teleostei: Gobiidae) inferred from mitochondrial and nuclear genes. Mar. Biol..

[22] Sharma A., Siva C., Ali S., Sahoo P.K., Nath R., Laskar M.A., Sarma D. (2020). The complete mitochondrial genome of the medicinal fish, *Cyprinion semiplotum*: Insight into its structural features and phylogenetic implications. Int. J. Biol. Macromol..

[23] Zhu S.-R., Fu J.-J., Wang Q., Li J.-L. (2013). Identification of Channa species using the partial cytochrome c oxidase subunit I (*COI*) gene as a DNA barcoding marker. Biochem. Syst. Ecol..

[24] Serrao N.R., Steinke D., Hanner R.H. (2014). Calibrating Snakehead Diversity with DNA Barcodes: Expanding Taxonomic Coverage to Enable Identification of Potential and Established Invasive Species. PLoS ONE.

[25] Rehman A., Khan M.F., Bibi S., Nouroz F. (2020). Comparative phylogeny of (*Schizothorax esocinus*) with reference to 12s and 16 sribosomal RNA from River Swat, Pakistan. Mitochondrial DNA Part A.

[26] Phan G.H., Lam T.T.H., Dinh Q.M., Nguyen T.H.D. (2023). Phylogenics of the genus *Glossogobius* in the Mekong Delta based on the mitochondrial cytochrome b (*cytb*) gene. Heliyon.

[27] Chandhini S., Vargheese S., Philip S., Rejish Kumar V.J. (2020). Deciphering the mitochondrial genome of Malabar snakehead, *Channa diplogramma* (Teleostei; Channidae). Biologia.

[28] Xu W., Lin S., Liu H. (2021). Mitochondrial genomes of five *Hyphessobrycon* tetras and their phylogenetic implications. Ecol. Evol..

[29] Zhang R., Zhu T., Luo Q. (2023). The Complete Mitochondrial Genome of the Freshwater Fish *Onychostoma ovale* (Cypriniformes, Cyprinidae): Genome Characterization and Phylogenetic Analysis. Genes.

[30] Liao X., Shih Y., Jia C., Gao T. (2024). Complete Mitochondrial Genome of Four Peristediidae Fish Species: Genome Characterization and Phylogenetic Analysis. Genes.

[31] Kajitani R., Toshimoto K., Noguchi H., Toyoda A., Ogura Y., Okuno M., Yabana M., Harada M., Nagayasu E., Maruyama H. (2014). Efficient de novo assembly of highly heterozygous genomes from whole-genome shotgun short reads. Genome Res..

[32] Dierckxsens N., Mardulyn P., Smits G. (2017). NOVOPlasty: *De novo* assembly of organelle genomes from whole genome data. Nucleic Acids Res..

[33] Iwasaki W., Fukunaga T., Isagozawa R., Yamada K., Maeda Y., Satoh T.P., Sado T., Mabuchi K., Takeshima H., Miya M. (2013). MitoFish and MitoAnnotator: A Mitochondrial Genome Database of Fish with an Accurate and Automatic Annotation Pipeline. Mol. Biol. Evol..

[34] Xiang C., Gao F., Jakovlić I., Lei H., Hu Y., Zhang H., Zou H., Wang G., Zhang D. (2023). Using PhyloSuite for molecular phylogeny and tree-based analyses. iMeta.

[35] Rozas J., Ferrer-Mata A., Sánchez-DelBarrio J.C., Guirao-Rico S., Librado P., Ramos-Onsins S.E., Sánchez-Gracia A. (2017). DnaSP 6: DNA Sequence Polymorphism Analysis of Large Data Sets. Mol. Biol. Evol..

[36] Katoh K., Standley D.M. (2013). MAFFT multiple sequence alignment software version 7: Improvements in performance and usability. Mol. Biol. Evol..

[37] Talavera G., Castresana J. (2007). Improvement of Phylogenies after Removing Divergent and Ambiguously Aligned Blocks from Protein Sequence Alignments. Syst. Biol..

[38] Nguyen L.T., Schmidt H.A., von Haeseler A., Minh B.Q. (2015). IQ-TREE: A fast and effective stochastic algorithm for estimating maximum-likelihood phylogenies. Mol. Biol. Evol..

[39] Ronquist F., Teslenko M., Van Der Mark P., Ayres D.L., Darling A., Höhna S., Larget B., Liu L., Suchard M.A., Huelsenbeck J.P. (2012). MrBayes 3.2: Efficient Bayesian Phylogenetic Inference and Model Choice Across a Large Model Space. Syst. Biol..

[40] Qin Q., Chen L., Zhang F., Xu J., Zeng Y. (2024). Characterization of the Complete Mitochondrial Genome of *Schizothorax kozlovi* (Cypriniformes, Cyprinidae, Schizothorax) and Insights into the Phylogenetic Relationships of *Schizothorax*. Animals.

[41] Miya M., Takeshima H., Endo H., Ishiguro N.B., Inoue J.G., Mukai T., Satoh T.P., Yamaguchi M., Kawaguchi A., Mabuchi K. (2003). Major patterns of higher teleostean phylogenies: A new perspective based on 100 complete mitochondrial DNA sequences. Mol. Phylogenet. Evol..

[42] Consuegra S., John E., Verspoor E., De Leaniz C.G. (2015). Patterns of natural selection acting on the mitochondrial genome of a locally adapted fish species. Genet. Sel. Evol..

[43] Ruan H., Li M., Li Z., Huang J., Chen W., Sun J., Liu L., Zou K. (2020). Comparative Analysis of Complete Mitochondrial Genomes of Three *Gerres* Fishes (Perciformes: Gerreidae) and Primary Exploration of Their Evolution History. Int. J. Mol. Sci..

[44] Lobry J.R. (1995). Properties of a general model of DNA evolution under no-strand-bias conditions. J. Mol. Evol..

[45] Sueoka N. (1995). Intrastrand Parity Rules of DNA Base Composition and Usage Biases of Synonymous Codons. J. Mol. Evol..

[46] Bielawski J.P., Gold J.R. (2002). Mutation patterns of mitochondrial H- and L-strand DNA in closely related Cyprinid fishes. Genetics.

[47] Colín A., Del Río-Portilla M.A., Lafarga-De La Cruz F., Ingle-De La Mora G., García-De León F.J. (2023). Assembly, Characterization, and Phylogenetic Relationships of Mitogenomes of Two Species of Mexican Trout (*Oncorhynchus chrysogaster* and *O. mykiss nelsoni*). Fishes.

[48] Satoh T.P., Miya M., Mabuchi K., Nishida M. (2016). Structure and variation of the mitochondrial genome of fishes. BMC Genom..

[49] Zhang R., Zhu T., Yu F. (2023). The New Mitochondrial Genome of *Hemiculterella wui* (Cypriniformes, Xenocyprididae): Sequence, Structure, and Phylogenetic Analyses. Genes.

[50] Zhang R., Zhu T., Li H., Deng L. (2023). The Mitochondrial Genome of *Linichthys laticeps* (Cypriniformes: Cyprinidae): Characterization and Phylogeny. Genes.

[51] Hogan R.I., Hopkins K., Wheeler A.J., Allcock A.L., Yesson C. (2019). Novel diversity in mitochondrial genomes of deep-sea Pennatulacea (Cnidaria: Anthozoa: Octocorallia). Mitochondrial DNA Part A.

[52] Shadel G.S., Clayton D.A. (1997). Mitochondrial DNA maintenance in vertebrates. Annu. Rev. Biochem..

[53] Liu C.C., Simonsen C., Levinson A. (1984). Initiation of translation at internal AUG codons in mammalian cells. Nature.

[54] Wang J., Xu W., Liu Y., Bai Y., Liu H. (2023). Comparative mitochondrial genomics and phylogenetics for species of the snakehead genus *Channa* Scopoli, 1777 (Perciformes: Channidae). Gene.

[55] Ojala D., Montoya J., Attardi G. (1981). tRNA punctuation model of RNA processing in human mitochondria. Nature.

[56] Efremov R.G., Baradaran R., Sazanov L.A. (2010). The architecture of respiratory complex I. Nature.

[57] Morio A., Tsutsumi R., Kondo T., Miyoshi H., Kato T., Narasaki S., Satomi S., Nakaya E., Kuroda M., Sakaue H. (2021). Leucine induces cardioprotection in vitro by promoting mitochondrial function via mTOR and Opa-1 signaling. Nutr. Metab. Cardiovasc. Dis..

[58] Zhao J., Zhao Y., Liu H., Cao Q., Feng L., Zhang Z., Jiang W., Wu P., Liu Y., Luo W. (2023). Dietary Leucine Improves Fish Intestinal Barrier Function by Increasing Humoral Immunity, Antioxidant Capacity, and Tight Junction. Int. J. Mol. Sci..

[59] Ben Slimen H., Awadi A., Tolesa Z.G., Knauer F., Alves P.C., Makni M., Suchentrunk F. (2018). Positive selection on the mitochondrial *ATP synthase 6* and the *NADH dehydrogenase 2* genes across 22 hare species (genus *Lepus*). J. Zool. Syst. Evol. Res..

[60] Li X., Huang Y., Lei F. (2015). Comparative mitochondrial genomics and phylogenetic relationships of the *Crossoptilon* species (Phasianidae, Galliformes). BMC Genom..

[61] Androsiuk P., Paukszto Ł., Jastrzębski J.P., Milarska S.E., Okorski A., Pszczółkowska A. (2022). Molecular Diversity and Phylogeny Reconstruction of Genus *Colobanthus* (Caryophyllaceae) Based on Mitochondrial Gene Sequences. Genes.

[62] Bazin E., Glémin S., Galtier N. (2006). Population size does not influence mitochondrial genetic diversity in animals. Science.

[63] Cheng Y., Wang R., Sun Y., Xu T. (2012). The Complete Mitochondrial Genome of the Small Yellow Croaker and Partitioned Bayesian Analysis of Sciaenidae Fish Phylogeny. Genet. Mol. Biol..

