# Supplementary material for: Characterization of Complete Mitochondrial Genome and Phylogeny of Three Echeneidae Species
Source: Animals (Basel). 2025 Jan 2;15(1):81. doi: 10.3390/ani15010081 (PMC11718899; doi:10.3390/ani15010081)
Supplement: Supplementary file 1 [file animals-15-00081-s001.zip › animals-3336687-supplementary.pdf]

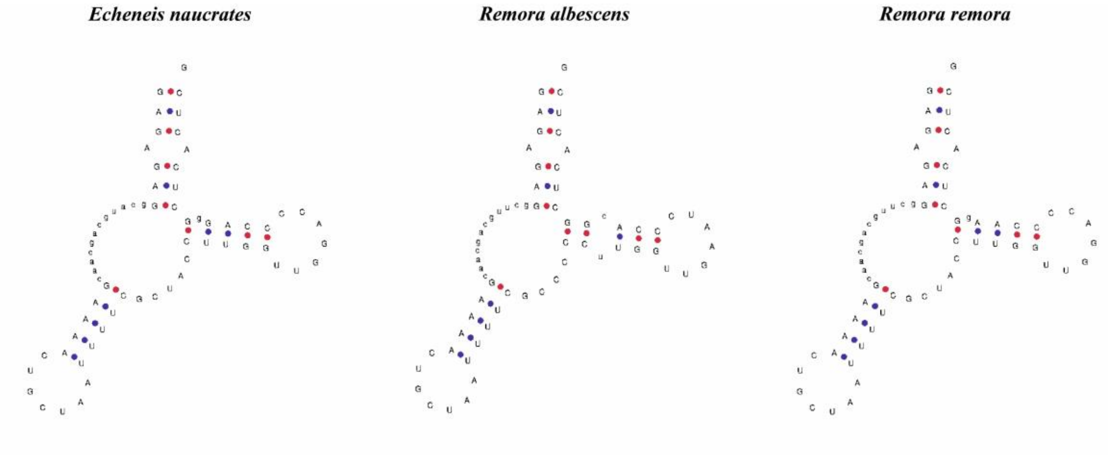

Figure S1. Predicted secondary structures of tRNA-Ser (GCT) gene in the mitochondrial genome of *E. naucrates*, *R. albescens*, and *R. remora*.

Table S1. Mitochondrial genome composition and characteristics of *E. naucrates.*

Gene	Position-start	Position-end	Length (bp)	Amino acid	Start codon	Stop codon	Strand
tRNA-Phe	1	68	68				H
12S RNA	69	1016	948				H
tRNA-Val	1017	1088	72				H
16S RNA	1089	2793	1705				H
tRNA-Leu	2794	2868	75				H
nd1	2869	3843	975	324	ATG	TAA	H
tRNA-Ile	3849	3918	70				H
tRNA-Gln	3917	3987	71				L
tRNA-Met	3987	4055	69				H
nd2	4056	5101	1046	348	ATG	TA	H
tRNA-Trp	5102	5171	70				H
tRNA-Ala	5173	5241	69				L
tRNA-Asn	5243	5315	73				L
tRNA-Cys	5353	5417	65				L
tRNA-Tyr	5418	5487	70				L
coxI	5489	7039	1551	516	GTG	TAA	H
tRNA-Ser	7040	7110	71				L
tRNA-Asp	7114	7181	68				H
coxII	7188	7878	691	230	ATG	T	H
tRNA-Lys	7879	7954	76				H
atpase8	7956	8123	168	55	ATG	TAA	H
atpase6	8114	8796	683	227	ATG	TA	H
coxIII	8797	9581	785	261	ATG	TA	H
tRNA-Gly	9582	9652	71				H
nd3	9653	10001	349	116	ATG	T	H
tRNA-Arg	10002	10070	69				H
nd4L	10071	10367	297	98	ATG	TAA	H
nd4	10361	11741	1381	460	ATG	T	H
tRNA-His	11742	11810	69				H
tRNA-Ser	11811	11877	67				H
tRNA-Leu	11883	11955	73				H
nd5	11956	13794	1839	611	ATG	TAA	H
nd6	13791	14312	522	173	ATG	TAA	L
tRNA-Glu	14313	14381	69				L
cytb	14387	15527	1141	380	ATG	T	H
tRNA-Thr	15528	15600	73				H
tRNA-Pro	15601	15671	71				L
D-loop	15672	16611	940				H

Table S2. Mitochondrial genome composition and characteristics of *R. albescens*.

Gene	Position-start	Position-end	Length (bp)	Amino acid	Start codon	Stop codon	Strand
tRNA-Phe	1	68	68				H
12S RNA	69	1015	947				H
tRNA-Val	1016	1087	72				H
16S RNA	1088	2799	1712				H
tRNA-Leu	2800	2874	75				H
nd1	2875	3849	975	324	ATG	TAA	H
tRNA-Ile	3858	3927	70				H
tRNA-Gln	3926	3996	71				L
tRNA-Met	3996	4064	69				H
nd2	4065	5110	1046	348	ATG	TA	H
tRNA-Trp	5111	5184	74				H
tRNA-Ala	5186	5254	69				L
tRNA-Asn	5256	5328	73				L
tRNA-Cys	5365	5429	65				L
tRNA-Tyr	5430	5499	70				L
coxI	5501	7051	1551	516	GTG	TAA	H
tRNA-Ser	7052	7122	71				L
tRNA-Asp	7126	7194	69				H
coxII	7201	7891	691	230	ATG	T	H
tRNA-Lys	7892	7966	75				H
atpase8	7968	8135	168	55	ATG	TAA	H
atpase6	8126	8808	683	227	ATG	TA	H
coxIII	8809	9593	785	261	ATG	TA	H
tRNA-Gly	9594	9665	72				H
nd3	9666	10014	349	116	ATG	T	H
tRNA-Arg	10015	10083	69				H
nd4L	10084	10380	297	98	ATG	TAA	H
nd4	10374	11754	1381	460	ATG	T	H
tRNA-His	11755	11823	69				H
tRNA-Ser	11824	11890	67				H
tRNA-Leu	11897	11969	73				H
nd5	11970	13808	1839	611	ATG	TAA	H
nd6	13805	14326	522	173	ATG	TAA	L
tRNA-Glu	14327	14395	69				L
cytb	14400	15540	1141	380	ATG	T	H
tRNA-Thr	15541	15612	72				H
tRNA-Pro	15614	15684	71				L
D-loop	15685	16648	964				H

Table S3. Mitochondrial genome composition and characteristics of *R. remora.*

Gene	Position-start	Position-end	Length (bp)	Amino acid	Start codon	Stop codon	Strand
tRNA-Phe	1	68	68				H
12S RNA	69	1018	950				H
tRNA-Val	1019	1090	72				H
16S RNA	1091	2797	1707				H
tRNA-Leu	2798	2872	75				H
nd1	2873	3847	975	324	ATG	TAA	H
tRNA-Ile	3858	3927	70				H
tRNA-Gln	3926	3996	71				L
tRNA-Met	3996	4064	69				H
nd2	4065	5110	1046	348	ATG	TA	H
tRNA-Trp	5111	5183	73				H
tRNA-Ala	5185	5253	69				L
tRNA-Asn	5255	5327	73				L
tRNA-Cys	5365	5429	65				L
tRNA-Tyr	5430	5499	70				L
coxI	5501	7051	1551	516	GTG	TAA	H
tRNA-Ser	7052	7122	71				L
tRNA-Asp	7126	7194	69				H
coxII	7200	7890	691	230	ATG	T	H
tRNA-Lys	7891	7965	75				H
atpase8	7967	8134	168	55	ATG	TAA	H
atpase6	8125	8807	683	227	ATG	TA	H
coxIII	8808	9592	785	261	ATG	TA	H
tRNA-Gly	9593	9663	71				H
nd3	9664	10012	349	116	ATG	T	H
tRNA-Arg	10013	10081	69				H
nd4L	10082	10378	297	98	ATG	TAA	H
nd4	10372	11752	1381	460	ATG	T	H
tRNA-His	11753	11821	69				H
tRNA-Ser	11822	11888	67				H
tRNA-Leu	11893	11965	73				H
nd5	11966	13804	1839	611	ATG	TAA	H
nd6	13801	14322	522	173	ATG	TAG	L
tRNA-Glu	14323	14391	69				L
cytb	14396	15536	1141	380	ATG	T	H
tRNA-Thr	15537	15609	73				H
tRNA-Pro	15610	15680	71				L
D-loop	15681	16623	943				H

Table S4. Sequence of the mitochondrial genome intergenic region in three species.

Gene	Species	Intergenic region (bp)	Sequences (5'-3')
	E. naucrates	5	AGACT
nd1-tRNA-Ile	*R. albescens*	8	TGCCCTCC
	R. remora	10	TGCCTTCCCC
	E. naucrates	-2	CT
tRNA-Ile-tRNA-Gln	*R. albescens*	-2	CT
	R. remora	-2	CT
	E. naucrates	-1	A
tRNA-Gln-tRNA-Met	*R. albescens*	-1	A
	R. remora	-1	A
	E. naucrates	1	A
tRNA-Trp-tRNA-Ala	*R. albescens*	1	A
	R. remora	1	A
	E. naucrates	1	C
tRNA-Ala-tRNA-Asn	*R. albescens*	1	A
	R. remora	1	G
	E. naucrates	37	CTTTCCCCCGCCTGTAAGTATTTACAAAGGCGGGGGA
tRNA-Asn- tRNA-Cys	*R. albescens*	36	CTTTCCCCCGCCTATAAATATACACAAAGGCGGGGG
	R. remora	37	CTTTCCCCCGCCTGTTAGTAGACACAAAGGCGGGGGA
	E. naucrates	1	T
tRNA-Tyr-*coxI*	*R. albescens*	1	T
	R. remora	1	T
	E. naucrates	3	TAT
tRNA-Ser-tRNA-Asp	*R. albescens*	3	CAT
	R. remora	3	TAT
	E. naucrates	6	AAAATT
tRNA-Asp-*coxII*	*R. albescens*	6	AAACTC
	R. remora	5	AACTT
	E. naucrates	1	C
tRNA-Lys-*atpase8*	*R. albescens*	1	T
	R. remora	1	C
	E. naucrates	-10	ATGACACTAA
atpase8-atpase6	*R. albescens*	-10	ATGACATTAA
	R. remora	-10	ATGACATTAA
	E. naucrates	-7	ATGCTAA
nd4L-nd4	*R. albescens*	-7	ATGCTAA
	R. remora	-7	ATGCTAA
	E. naucrates	5	TATAA
tRNA-Ser-tRNA-Leu	*R. albescens*	6	TCCAAA
	R. remora	4	TAGG
nd5-nd6	*E. naucrates*	-4	TTAA

	R. albescens	-4	TTAA
	R. remora	-4	CTAA
	E. naucrates	5	ACTTA
tRNA-Glu-*cytb*	*R. albescens*	4	ACTA
	R. remora	4	ACTT
tRNA-Pro	*R. albescens*	1	A

Table S5. The base composition of *E. naucrates* mitochondrial genome.

	Size (bp)	T%	C%	A%	G%	A+T%	C+G%	AT skewness	GC skewness
Mitogenome	16611	29.3	25.4	30.3	15.0	59.6	40.4	0.016	-0.257
atpase6	683	32.5	26.8	29.0	11.7	61.5	38.5	-0.057	-0.392
atpase8	168	29.8	29.2	32.7	8.3	62.5	37.5	0.048	-0.556
coxI	1551	33.8	22.2	26.4	17.5	60.2	39.7	-0.123	-0.117
coxII	691	30.5	24.7	28.4	16.4	58.9	41.1	-0.037	-0.204
coxIII	785	29.7	27.0	26.1	17.2	55.8	44.2	-0.064	-0.222
cytb	1141	31.1	26.5	28.0	14.4	59.1	40.9	-0.052	-0.296
nd1	975	30.9	29.2	26.3	13.6	57.2	42.8	-0.081	-0.364
nd2	1046	28.3	31.3	30.6	9.8	58.9	41.1	0.039	-0.521
nd3	349	33.2	26.4	25.8	14.6	59.0	41.0	-0.126	-0.287
nd4	1381	31.9	26.1	29.6	12.5	61.5	38.6	-0.037	-0.353
nd4L	297	32.3	28.3	27.3	12.1	59.6	40.4	-0.085	-0.4
nd5	1839	31.3	27.6	29.3	11.9	60.6	39.5	-0.032	-0.399
nd6	522	42.5	10.5	20.7	26.2	63.2	36.7	-0.345	0.427
rRNA	2653	22.8	22.7	34.7	19.8	57.5	42.5	0.206	-0.068
tRNA	1549	27.8	20.6	28.9	22.8	56.7	43.4	0.019	0.051
PCGs	11418	31.8	26.0	27.9	14.3	59.7	40.3	-0.066	-0.292
D-loop	940	33.9	19.5	34.0	12.6	67.9	32.1	0.001	-0.215

Table S6. The base composition of *R. albescens* mitochondrial genome.

	Size (bp)	T%	C%	A%	G%	A+T%	C+G%	AT skewness	GC skewness
Mitogenome	16648	28.1	27.6	28.7	15.6	56.8	43.2	0.012	-0.276
atpase6	683	30.5	30.6	27.1	11.9	57.6	42.5	-0.059	-0.441
atpase8	168	29.2	29.2	33.3	8.3	62.5	37.5	0.067	-0.556
coxI	1551	31.5	25.0	24.8	18.7	56.3	43.7	-0.118	-0.145
coxII	691	29.4	25.3	28.2	17.1	57.6	42.4	-0.02	-0.195
coxIII	785	29.0	28.7	25.5	16.8	54.5	45.5	-0.065	-0.261
cytb	1141	30.2	30.5	25.0	14.3	55.2	44.8	-0.095	-0.362
nd1	975	30.1	30.6	25.2	14.2	55.3	44.8	-0.087	-0.367
nd2	1046	27.2	34.7	26.4	11.7	53.6	46.4	-0.016	-0.497
nd3	349	32.4	30.4	23.2	14.0	55.6	44.4	-0.165	-0.368
nd4	1381	29.3	29.2	28.5	13.0	57.8	42.2	-0.013	-0.383
nd4L	297	31.0	32.3	21.9	14.8	52.9	47.1	-0.172	-0.371
nd5	1839	29.6	29.6	28.1	12.7	57.7	42.3	-0.027	-0.398
nd6	522	38.7	12.8	18.4	30.1	57.1	42.9	-0.356	0.402
rRNA	2659	22.6	24.0	33.0	20.4	55.6	44.4	0.187	-0.082
tRNA	1553	27.0	21.3	29.0	22.7	56.0	44.0	0.036	0.032
PCGs	11418	30.2	28.6	26.1	15.1	56.3	43.7	-0.073	-0.31
D-loop	964	33.5	19.5	34.1	12.9	67.6	32.4	0.009	-0.204

Table S7. The base composition of *R. remora* mitochondrial genome.

	Size (bp)	T%	C%	A%	G%	A+T%	C+G%	AT skewness	GC skewness
Mitogenome	16623	28.4	27.2	28.3	16.2	56.7	43.4	-0.002	-0.253
atpase6	683	30.7	29.6	27.8	11.9	58.5	41.5	-0.05	-0.428
atpase8	168	29.8	32.7	27.4	10.1	57.2	42.8	-0.042	-0.528
coxI	1551	31.8	24.4	25.1	18.7	56.9	43.1	-0.118	-0.133
coxII	691	30.2	24.3	28.9	16.5	59.1	40.8	-0.022	-0.191
coxIII	785	29.0	27.1	26.8	17.1	55.8	44.2	-0.041	-0.228
cytb	1141	30.4	30.0	24.7	14.9	55.1	44.9	-0.103	-0.336
nd1	975	30.3	29.8	23.8	16.1	54.1	45.9	-0.12	-0.299
nd2	1046	29.3	32.7	27.3	10.7	56.6	43.4	-0.034	-0.507
nd3	349	33.5	28.9	21.2	16.3	54.7	45.2	-0.225	-0.278
nd4	1381	29.6	29.0	27.5	13.9	57.1	42.9	-0.037	-0.351
nd4L	297	31.6	31.6	23.6	13.1	55.2	44.7	-0.146	-0.414
nd5	1839	29.5	30.1	26.6	13.7	56.1	43.8	-0.051	-0.375
nd6	522	37.2	13.4	20.1	29.3	57.3	42.7	-0.298	0.372
rRNA	2657	22.7	23.7	32.7	20.9	55.4	44.6	0.182	-0.065
tRNA	1552	26.5	21.6	28.0	23.9	54.5	45.5	0.026	0.051
PCGs	11418	30.5	28.1	25.8	15.5	56.3	43.6	-0.083	-0.29
D-loop	943	33.0	19.8	34.1	13.0	67.1	32.8	0.016	-0.207
